# Study on the metabolic changes and regulatory mechanism of *Aspergillus flavus* conidia germination

**DOI:** 10.1128/spectrum.00108-24

**Published:** 2024-07-23

**Authors:** Sifan Jia, Chong Li, Yu An, Desheng Qi

**Affiliations:** 1 Department of Animal Nutrition and Feed Science, College of Animal Science and Technology, Huazhong Agricultural University, Wuhan, Hubei, China; University of Mississippi, University, Mississippi, USA

**Keywords:** *Aspergillus flavus*, conidia germination, carbon metabolism, nitrogen metabolism

## Abstract

*Aspergillus flavus* conidia are widespread in air; they attach to food and feed crops and secrete aflatoxins, which results in serious contamination. Germination of *A. flavus* conidia is the most critical step in contamination of food by *A. flavus*. This study aims to gain an insight into *A. flavus* conidia through dormancy to germination to provide a theoretical basis for inhibition of *A. flavus* conidia germination. The morphological changes and regulation mechanism of *A. flavus* conidia germination at 0, 4, 8, and 12 hours were observed. Transcriptomic and metabolomic analyses showed that conidia became active from dormancy (0 hour) to the initial stage of germination (4 hours), cellular respiration and energy metabolism increased, and amino acids and lipids were synthesized rapidly. The number of differentially expressed genes and differential metabolites was highest at this stage. Besides, we found that conidia germination had selectivity for different carbon and nitrogen sources. Compared with monosaccharides, disaccharides, as the only carbon source, significantly promoted the germination of conidia. Moreover, *MepA*, one of genes in the ammonium transporter family was studied. The gene deletion mutant *ΔMepA* had a significant growth defect, and the expression of *MeaA* was significantly upregulated in *ΔMepA* compared with the wild-type, indicating that both *MepA* and *MeaA* played an important role in transporting ammonium ions.

**IMPORTANCE:**

This is the first study to use combined transcriptomic and metabolomics analyses to explore the biological changes during germination of *Aspergillus flavus* conidia. The biological process with the highest changes occurred in 0–4 hours at the initial stage of germination. Compared with polysaccharides, monosaccharides significantly increased the size of conidia, while significantly decreasing the germination rate of conidia. Both MeaA and MepA were involved in ammonia transport and metabolism during conidia germination.

## INTRODUCTION

Aflatoxins are secondary metabolites mainly secreted by *Aspergillus flavus* and *Aspergillus parasiticus* during the growth process (1).They contaminate food and its raw materials, have strong teratogenic and mutagenic effects, and are listed as carcinogens (2). Aflatoxins could cause chronic toxicity in humans and animals via long-term and low-dose intake and may induce liver cancer in severe cases; they could also cause acute toxicity or even death by a short-term high-dose intake (3).

Conidia are one of the most common bioaerosols; they may attach to food and feed crops and germinate under suitable conditions (4). The *A. flavus* genome sequence contains 13,071 genes (5), including a 70-kb gene cluster with 24 structural genes involved in the aflatoxin biosynthetic pathway, regulated by *aflR* and *aflS* genes (6, 7). *Aspergillus* conidia germination involves a series of complex morphological changes that allow the fungus to grow in a suitable condition. Conidia germination is activated by the presence of water and nutrients, which triggers a series of morphological changes (8). One of the early stages of *Aspergillus* conidia germination is swelling. This occurs due to the absorption of water, which causes the conidia to increase in size and swell. Swelling is necessary for conidia to undergo the subsequent morphological changes and begin the germination process. The second step in conidia germination is the formation of a germ tube. The germ tube is essential for the conidia to establish contact with the substrate and absorb nutrients. It also serves as the point of attachment for the developing hyphae, which are the main structures of the fungus. During this period, the cytoskeleton, vesicle trafficking system, and signaling pathways were changed drastically (9). As the germ tube grows, it undergoes several morphological changes. It becomes thicker and more branched, and eventually, a primary hypha emerges. This primary hypha is the first structure of the fungus that can actively grow and penetrate the substrate (10). It was revealed that proteins related to MAPK signaling pathway played an important role in *A. flavus* conidia germination. Also, proteins connected to cell wall synthesis and degradation, metabolism, protein synthesis, and degradation were essential (11). Germination of *A. flavus* conidia is the most critical step in *A. flavus* contamination of food (12). Therefore, exploring the biological mechanism of *A. flavus* conidia germination is conducive to a better understanding of the process and germination conditions, which may provide a theoretical basis for the inhibition of *A. flavus* conidia germination to reduce aflatoxin pollution.

Different foods have different degrees of mold contamination, which may be related to the composition of the food (13). Fungi have selectivity for different nutrients, such as carbon, nitrogen, and lipid sources (14). Also, research showed that during germination, aspergilli differ in competitive potential depending on the substrate (15). It was revealed that in the inducing medium, the germination of *A. niger* was heterogeneous. To germinate, *A. niger* conidia require a combination of an inducing carbon source and either inorganic phosphate, inorganic nitrogen, or magnesium sulfate (16). A previous study reported (17) that the high concentrations of internal carbohydrates in dormant conidia did not affect the function of proteins or membranes. There were significant differences in the content of various carbohydrates among different fungi. Research showed (18) that mannitol stored in conidia of *A. niger* accounted for 10%–15% of its dry weight, and the ratio of mannitol: trehalose in *A. oryzae* was about 3:1. Also, a study revealed that genes related to trehalose and mannitol biosynthesis were connected to heat sensitivity during *A. niger* conidia germination (19). It was reported (20) that the main carbohydrate in conidia of *A. nidulans* and *Neurospora crassa* was trehalose, and the content of trehalose in *A. nidulans* was 0.65–1.0 picograms per conidia, equivalent to 2%–4 % of the wet weight of conidia. The nitrogen source is the basic raw material of a variety of compounds related to *Aspergillus* growth, which is mainly divided into inorganic nitrogen sources and organic nitrogen sources. Nitrogen metabolism is one of the most important types of nutrient utilization and has a significant impact on the growth, reproduction, secondary metabolite synthesis (such as aflatoxin), and other phenotypes of fungi (21). Although there have been many studies (22, 23) on different carbon and nitrogen sources in the growth process of *Aspergillus*, few of them focused on the period of *A. flavus* conidia germination. Our study aimed to examine *A. flavus* conidia through dormancy to germination. To our knowledge, we are the first to combine both the transcriptome and metabolome to explore the mechanism of *A. flavus* conidia germination. A comprehensive understanding of *A. flavus* conidia germination could provide a theoretical basis for inhibition of *A. flavus* conidia germination to reduce aflatoxin pollution. Moreover, understanding the effects of different carbon and nitrogen sources on germination of *A. flavus* conidia could help us explore the causes of different levels of aflatoxin contamination in food and feed crops with different carbon and nitrogen components.

## MATERIALS AND METHODS

### Fungal strains

Two different *Aspergillus flavus* strains were used in this study. The *Aspergillus flavus* strain NRRL 3357 was kindly provided by Zhumei He (Sun Yat-sen University, Guangzhou, China). The *A. flavus ΔMepA* strain was constructed with fusion PCR (24), and the primer sequences are shown in Table S1. Conidia were cultured on Czapek–Dox medium at 30°C for 7 days, and then conidia were collected and suspended in sterile water (with 0.05% Tween-80). The conidia suspension was filtered through four layers of sterile lens paper and kept on ice until further processing. Conidia were counted by using a hemocytometer (25) and then diluted to 10^6^ per mL (dilution factor = total number of conidia per milliliter / 10^6^).

To determine the germination rate of *A. flavus* conidia, at least 200 conidia were examined in each sample for six repetitions. Conidia were regarded as germinated when at least 2 µm of the germ tube was visible.

### Culture conditions

For the morphological observation and flow cytometry, conidia were cultured with 100 mL Czapek–Dox medium at 30°C for 4 hours, 8 hours, and 12 hours. For the experiment of carbon metabolism, conidia were cultured with different carbon resources with equal atomic carbon. The Czapek–Dox medium was used to study sucrose (30 g/L) metabolism, and trehalose (30 g/L), fructose (57 g/L), and glucose (57 g/L) were used to replace the carbon source in the culture medium with other components unchanged. Conidia were cultured with 100 mL of the medium at 30°C for 4 hours, 8 hours, and 12 hours. For the experiment of nitrogen metabolism, conidia were cultured with different nitrogen resources with equal atomic nitrogen. The Czapek–Dox medium was used to study nitrate (3 g/L) metabolism, and nitrite (2.44 g/L) and ammonium (1.89 g/L) were used to replace the nitrogen source in the culture medium with other components unchanged. Conidia were cultured with 100 mL of the medium at 30°C for 8 hours and 12 hours.

### Scanning electron microscopy

The method of scanning electron microscopy used in this study was modified from that of Jia *et al*. (26). Conidia were inoculated into iquid Czapek–Dox medium at a concentration of 10^6^ per mL, with shaking at 150 rpm at 30°C for 4 hours, 8 hours, and 12 hours. Conidia were then harvested by centrifugation at 3,000 rpm for 5 minutes. Impurities were removed by washing the conidia twice with phosphate-buffered saline (PBS). The washed conidia were then fixed in 2.5% glutaraldehyde at 25°C for 2 hours and transferred to 4°C for preservation and transportation. Then, conidia were washed three times with 0.1M phosphate buffer (PB) (pH 7.4), 15 minutes each time. The washed conidia were then transferred into 1% OsO_4_ in 0.1 M PB (pH 7.4) for about 1–2 hours at 25°C. After these steps, the conidia were washed additionally three times in 0.1 M PB (pH 7.4), for 15 minutes each time. Then, gradient elution with ethanol was performed (concentration of 30%–100%), and conidia samples were dried with a critical point dryer. Specimens were attached to metallic stubs using carbon stickers and sputter-coated with gold for 30 seconds. Conidia were observed and images taken with a HITACHI Regulus 8100 scanning electron microscope (Tokyo, Japan).

### Flow cytometry of conidia

The method of flow cytometry of conidia in this study was modified from that of Hayer *et al*. (27). The size and complexity of conidia were measured by flow cytometry at 4 hours and 8 hours of germination. Conidia were inoculated into liquid Czapek–Dox medium at a concentration of 10^6^ per mL, with shaking at 150 rpm at 30°C for 4 hours and 8 hours. Conidia were then harvested by centrifugation at 3,000 rpm for 5 minutes. After that, the supernatant was removed and the conidia washed with 1 mL sterile water (containing 0.01% Tween-80). Flow cytometry (Beckman-CytoFLEX Coulter, CA, USA) was then used to analyze the forward scatter (FSC) parameter and side scatter (SSC) of conidia.

### RNA-Seq analysis

The methods of RNA-seq analysis in this study were modified from that of Jia *et al*. (26). Total RNA was extracted from 0-hour, 4-hour, 8-hour, and 12-hour conidia using TRIzol Reagent (plant rna purification reagent for plant tissue; Invitrogen), according to the manufacturer’s instructions. An RNA-seq transcriptome library was prepared following the TruSeqTM RNA sample preparation Kit from Illumina (San Diego, CA), using 1 µg of total RNA. The raw paired-end reads were trimmed and quality-controlled by SeqPrep (https://github.com/jstjohn/SeqPrep) and Sickle (https://github.com/najoshi/sickle) with default parameters. Then, clean reads were separately aligned to the reference genome in the orientation mode using HISAT2 software (https://daehwankimlab.github.io/hisat2/)) (28). The mapped reads of each sample were assembled with StringTie (https://ccb.jhu.edu/software/stringtie/) using a reference-based approach (29). To identify DEGs (differentially expressed genes), DESeq analysis was used. In transcriptomics analysis, differentially expressed genes were considered significant if the fold change was >2 and the *P*-value was  <0.05. In addition, functional enrichment analyses, including GO functional enrichment (30) and KEGG pathway analysis (31), were performed to identify the DEGs that were significantly enriched in GO terms and metabolic pathways by Fisher’s exact test at a Bonferroni-corrected *P*-value ≤ 0.05 compared with the whole-transcriptome background. GO functional enrichment and KEGG pathway analysis were carried out by Goatools (https://github.com/tanghaibao/Goatools) and KOBAS (http://kobas.cbi.pk-u.edu.cn/home.do) (32).

### Metabolomic analysis

The method of metabolomic analysis used in this study was modified from that of Wu *et al*. (33). Fresh conidia of 0, 4, 8, and 12 hours were harvested and freeze-dried in a vacuum freeze-dryer (Scientz-100F). The freeze-dried sample was crushed using a mixer mill (MM 400, Retsch) with a zirconia bead for 1.5 minutes at 30 Hz. One hundred milligrams of the lyophilized powder was dissolved in 1.2 mL 70% methanol solution, vortexed for 30 seconds every 30 minutes six times in total, and placed in a refrigerator at 4°C overnight. Following centrifugation at 12,000 rpm for 10 minutes, the extracts were filtered (SCAA-104, 0.22 µm pore size; ANPEL, Shanghai, China, http://www.anpel.com.cn/) before UPLC-MS/MS analysis. The sample extracts were analyzed using an UPLC-ESI-MS/MS system (UPLC, SHIMADZU Nexera X2, www.shimadzu.com.cn/; MS, Applied Biosystems 4500 Q TRAP, www.appliedbiosystems.com.cn/). The noise was removed from the raw data to smooth the waveforms, the data were baseline-corrected, and overlapping peaks were identified to analyze various metabolites (34). Significantly regulated metabolites between groups were determined by VIP >1 and absolute Log_2_FC (fold change) >1. VIP values were extracted from the OPLS-DA result, which also contained score plots and permutation plots, generated using the R package MetaboAnalystR. The data were log-transformed (log_2_) and mean-centered before OPLS-DA. In order to avoid overfitting, a permutation test (200 permutations) was performed. Identified metabolites were annotated using the KEGG compound database (https://www.kegg.jp/kegg/compound/). Annotated metabolites were then mapped to the KEGG Pathway database (http://www.kegg.jp/kegg/pathway.html). Pathways with significantly regulated metabolites mapped to them were then fed into MSEA (metabolite set enrichment analysis), and their significance was determined from the *P*-values of the hypergeometric test.

### Reverse transcription quantitative PCR (RT-qPCR)

In order to identify the transcriptomic data and to further investigate the function of these genes, several genes were selected to perform real-time quantitative PCR (qPCR). The total RNA was extracted with the same transcriptomic method, and the quality and amount of RNA were measured by using a Thermo NanoDrop (Thermo Fisher Scientific). The cDNA was synthesized from the extracted RNA using a PrimeScript RT reagent Kit (Takara). qPCR was conducted on a Bio-Rad CFX384 Real-Time PCR System with TB Green Premix Ex Taq II (Tli RNaseH Plus) (Takara, Dalian, China). The relative amounts of mRNAs were normalized with the housekeeping gene *GAPDH* and were analyzed by the 2–^ΔΔCt^ method (35). The sequences of the primers used in this study are shown in Table S2.

### Statistical analysis

The significance of conidia germination, flow cytometry, and qPCR data were analyzed using the SPSS Statistics 21.0 package (SPSS Inc., IBM, New York, NY, USA). One-way analysis of variance (ANOVA) was used to test for differential effects of the data. The figures were analyzed using GraphPad Prism 6 (Graph Pad Software Inc., San Diego, CA, USA) and processed using Adobe Illustrator CC 2019 (Adobe Systems Inc., San Jose, CA, USA). Flow cytometry data were analyzed using FlowJo_V10.

## RESULTS

### Conidia germination

**Fig 1:**
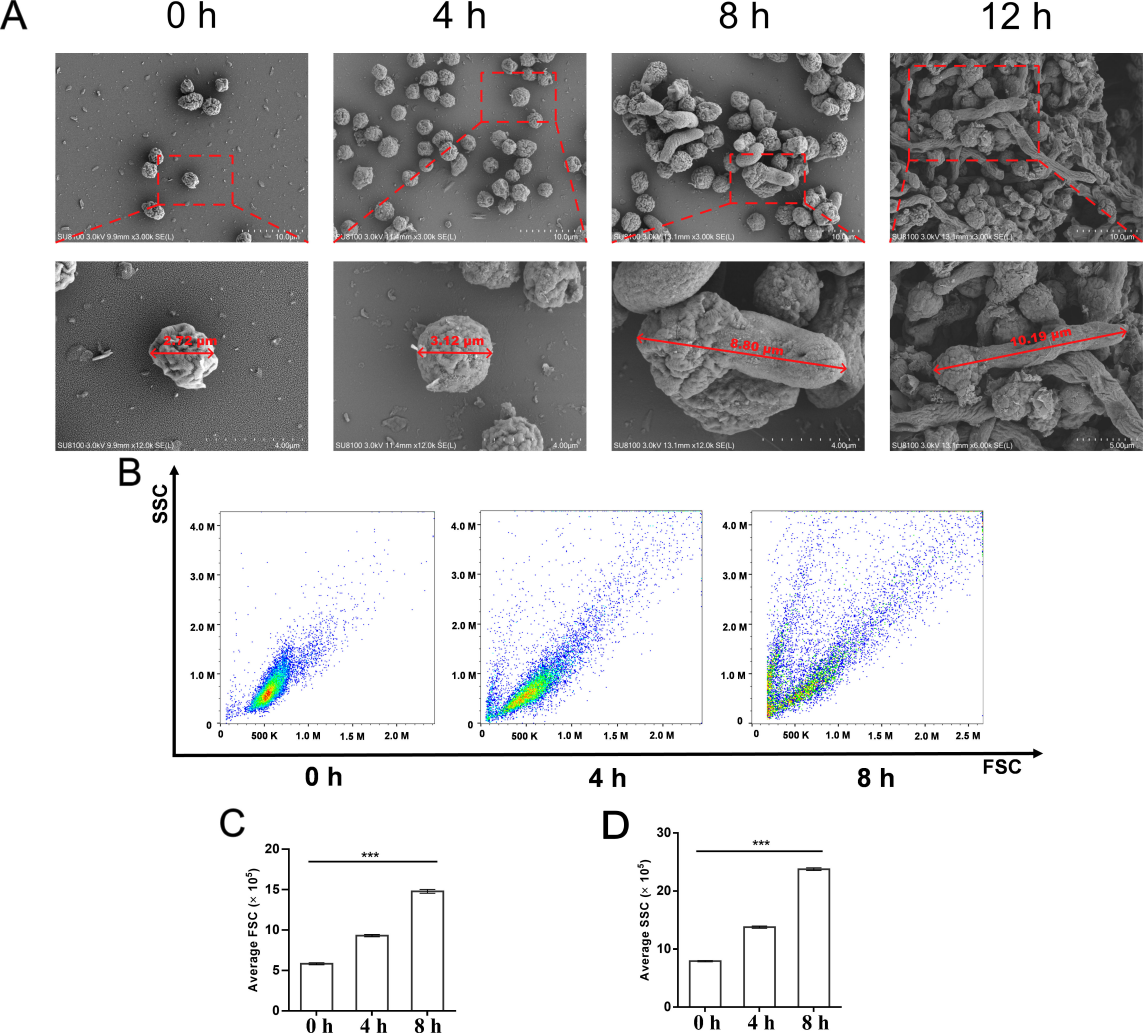
Morphological changes during *A. flavus* conidia germination. The stages of conidia at 0 hour, 4 hours, 8 hours, and 12 hours were observed by scanning electron microscopy (SEM) (A), and stages of conidia at 0 hour, 4 hours, and 8 hours were observed and analyzed by flow cytometry (B). The x-axis indicates forward scatter (FSC), and the y-axis indicates side scatter (SSC). C and D indicate average FSC and SSC parameters of 10,000 conidia, respectively; the means and standard errors of duplicate samples have been plotted (*n* = 3). The ‘***’ on the column diagram indicates a significant difference in the treatment at *P* < 0.001 in (C–D).

To depict the process of *A. flavus* conidia germination, scanning electron microscopy (SEM) analysis was performed at 0, 4, 8 and 12 hours to determine the dormant conidia, swelling (early stage), germ tube formation (middle stage), and mycelium formation (later stage). According to the SEM results (Fig. 1A), conidia were 2.72 µm in diameter at 0 hour, were found to be irregularly spherical, and many gullies on the surface were clearly visible. After culturing for 4 hours, conidia swelled significantly by absorbing water and increased to 3.12 µm in diameter, and the surface of the conidia became smooth compared with that of the dormant conidia. After culturing for 8 hours, some conidia began to exhibit polar growth, and at one side of the sphere, a germ tube appeared. The length of conidia was 8.80 µm. After culturing for 12 hours, a longer mycelium was formed, and the length of conidia was 10.19 µm. The sphere was still visible, and a small number of conidia grew into a second germ tube, gradually forming the mycelium.

The flow cytometry data are shown in Fig. 1B. It was revealed that the forward scatter (FSC) and side scatter (SSC) of conidia increased over time. To some extent, the FSC reflected the size of conidia, and the SSC reflected the complexity of conidia, which mean the proportion of conidia currently in different stages. As shown in Fig. 1C and D, at the early stage (4 hours) and middle stage (8 hours) of germination, the size of conidia increased significantly, and the cell complexity of conidia increased significantly after formation of the germ tube (8 h). Compared with the dormant stage, the size of conidia increased about 1.5 times after culturing for 4 hours, and the cell complexity increased significantly, indicating that the number of conidia in different stages increased; after 8 hours, the size increased by about 2.5 times, and the cell complexity increased significantly. The results showed that after culturing for 8 hours, the number of conidia at different stages gradually increased, and the rate of increase in conidia diversity gradually increased over time.

### Transcriptomic analysis

**Fig 2:**
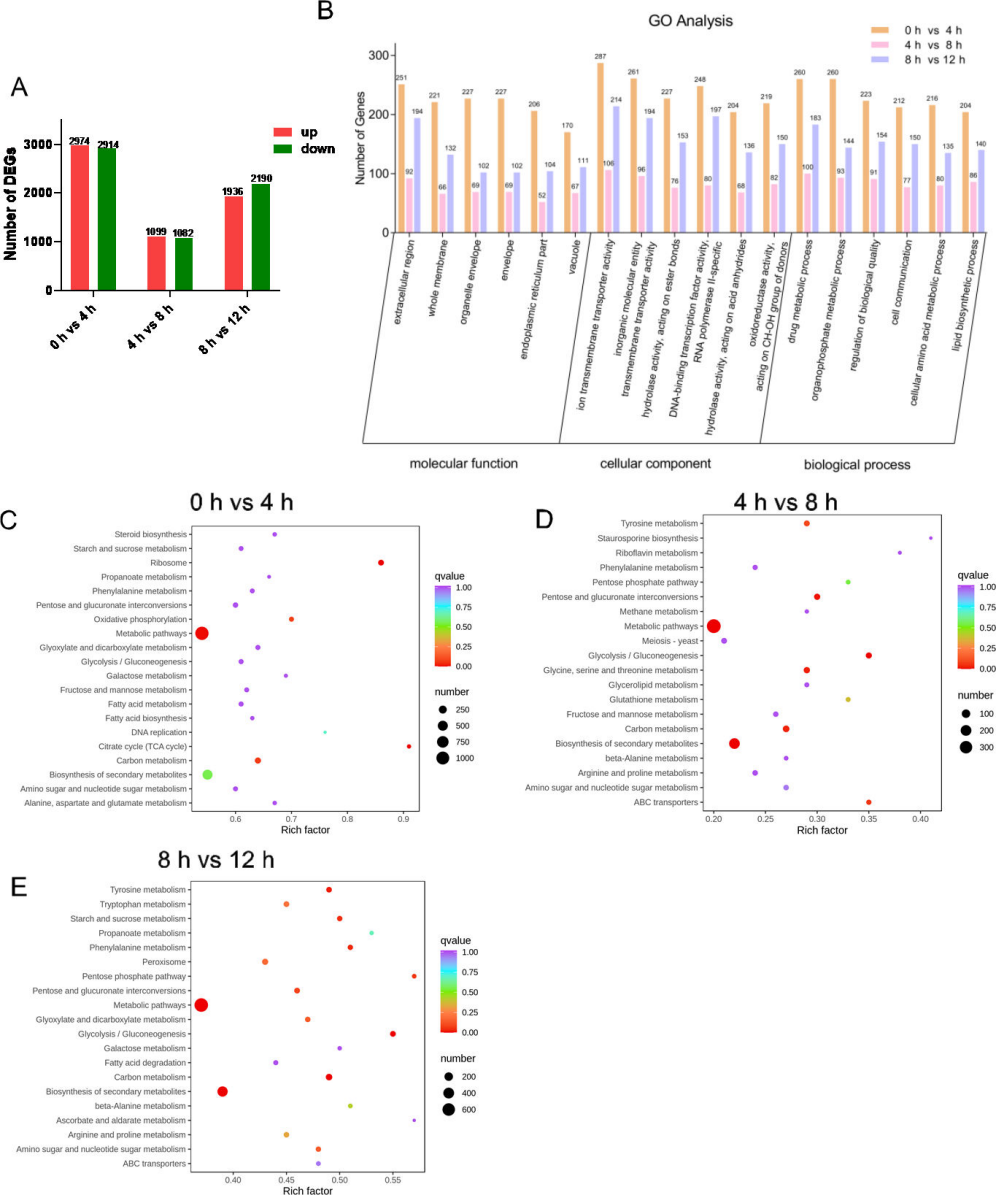
Summary of differentially regulated genes. The gene expression of two groups was significantly (*P* < 0.05) differentially expressed as fold change >2. (A) Number of differentially expressed genes (DEGs) in each group. (B) Top six of molecular function, cellular component, and biological process of Gene Ontology (GO) categorization in three independent groups. KEGG enrichment analysis of 0 hour vs 4 hours (C), 4 hours vs 8 hours (D), and 8 hours vs 12 hours (E) is represented. The x-axis indicates the Rich factor, and the y-axis indicates KEGG pathways; the size and color represent the number of genes enriched in the pathway and the level of pathway impact value, respectively.

The transcriptomic results showed (Fig. 2A) that the highest number of DEGs was observed at 0 hour vs 4 hours, with a total of 5,888 DEGs, including 2,914 downregulated genes and 2,974 upregulated genes; the second highest number of DEGs was observed at 8 hours vs 12 hours, with a total of 4,126 DEGs, including 1,936 upregulated genes and 2,190 downregulated genes; the lowest number of DEGs was observed at 4 hours vs 8 hours, with a total of 2,181 DEGs, including 1,099 upregulated genes and 1,082 downregulated genes. GO functional analysis was performed among these three groups (Fig. 2B), and it was demonstrated that the DEGs were mainly concentrated in the extracellular region, whole membrane, ion transmembrane transporter activity, inorganic molecular entity transmembrane transporter activity, drug metabolic process, and organophosphate metabolic process. Moreover, KEGG enrichment analysis was performed, and the top 20 pathways were listed depending on the Rich factor (Fig. 2C through E). At the initial stage of germination (0 hour vs 4 hours), the significantly different pathways were TCA cycle, ribosome, DNA replication, and so on; at the middle stage of germination (4 hours vs 8 hours), the significantly different pathways were staurosporine biosynthesis; riboflavin metabolism; glycine, serine, and threonine metabolism; and so on. At the later stage of germination (8 hours vs 12 hours), the significantly different pathways were ascorbate and aldarate metabolism, pentose phosphate pathway, glycolysis, and so on. Interested DEGs in KEGG pathways are shown in Table S3.

### Metabolomic analysis

**Fig 3:**
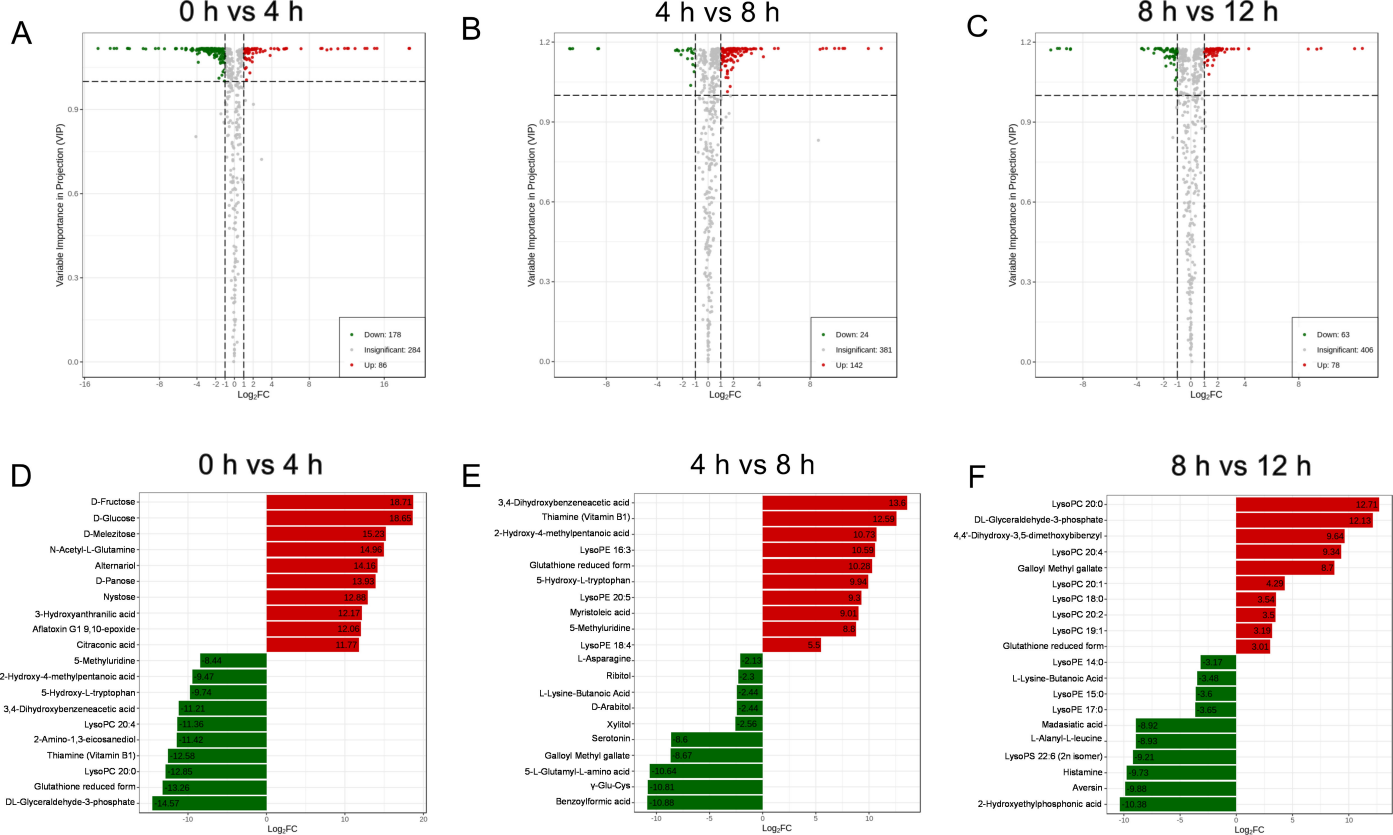
Summary of regulated metabolites. Metabolites with significant regulation between groups were determined by Variable Importance in Projection (VIP) > 1 and absolute Log_2_FC (fold change) >1. Volcano plots of significantly regulated metabolites at 0 hour vs 4 hours (**A**), 4 hours vs 8 hours (**B**), and 8 hours vs 12 hours (**C**) are represented. The red dots represent upregulated metabolites, the green dots represent downregulated metabolites, and the gray dots represent metabolites without significant regulation. The top 10 regulated metabolites at 0 hour vs 4 hours (**D**), 4 hour vs 8 hours (**E**), and 8 hours vs 12 hours (**F**) are shown. The red bar represents upregulated metabolites, and the green bar represents downregulated metabolites.

The differential metabolites identified in metabolomics analysis are shown in Fig. 3A through C. During the germination of *A. flavus* conidia, the differential metabolites with the highest number of changes were observed at 0 hour vs 4 hours, with a total of 264 differential metabolites, of which 86 were upregulated and 178 were downregulated; the second highest number of changes occurred between 4 hours and 8 hours, with a total of 166 differential metabolites, of which 142 were upregulated and 24 were downregulated; by contrast, the lowest number of changes was observed at 8 hours vs 12 hours, with a total of 141 differential metabolites, of which 78 were upregulated and 63 were downregulated. Qualitative and quantitative analyses of the detected metabolites were performed to compare multiple differences in metabolites among different groups. Figure 3D through F show the results of differential metabolites with differences across the top 20 after log_2_ transformation compared across groups: during the initial stage of germination (0 hour–4 hours), the greatest fold changes (FC) in metabolites were seen in D-fructose, D-glucose, and D-melezitose, and the most downregulated FC of metabolites was DL-glyceraldehyde-3-phosphate, glutathione reduced form, and lysoPC 20:0; during the middle stage of germination (4 hours–8 hours), the most upregulated FC of metabolites were 3,4-dihydroxybenzeneacetic acid, thiamine (vitamin B1), and 2-hydroxy-4-methylpentanoic acid, and the most downregulated FC of metabolites were benzoylformic acid, γ-Glu-Cys and 5-L-glutamyl-L-amino acid; and during the later stage of germination (8 hours–12 hours), the most upregulated FC of metabolites were lysoPC 20:0, DL-glyceraldehyde-3-phosphate, and 4,4′-dihydroxy-3,5-dimethoxybibenzyl, and the most downregulated FC of metabolites was 2-hydroxyethylphosphonic acid, aversin, and histamine.

### Combined transcriptomic and metabonomic analysis

**Fig 4:**
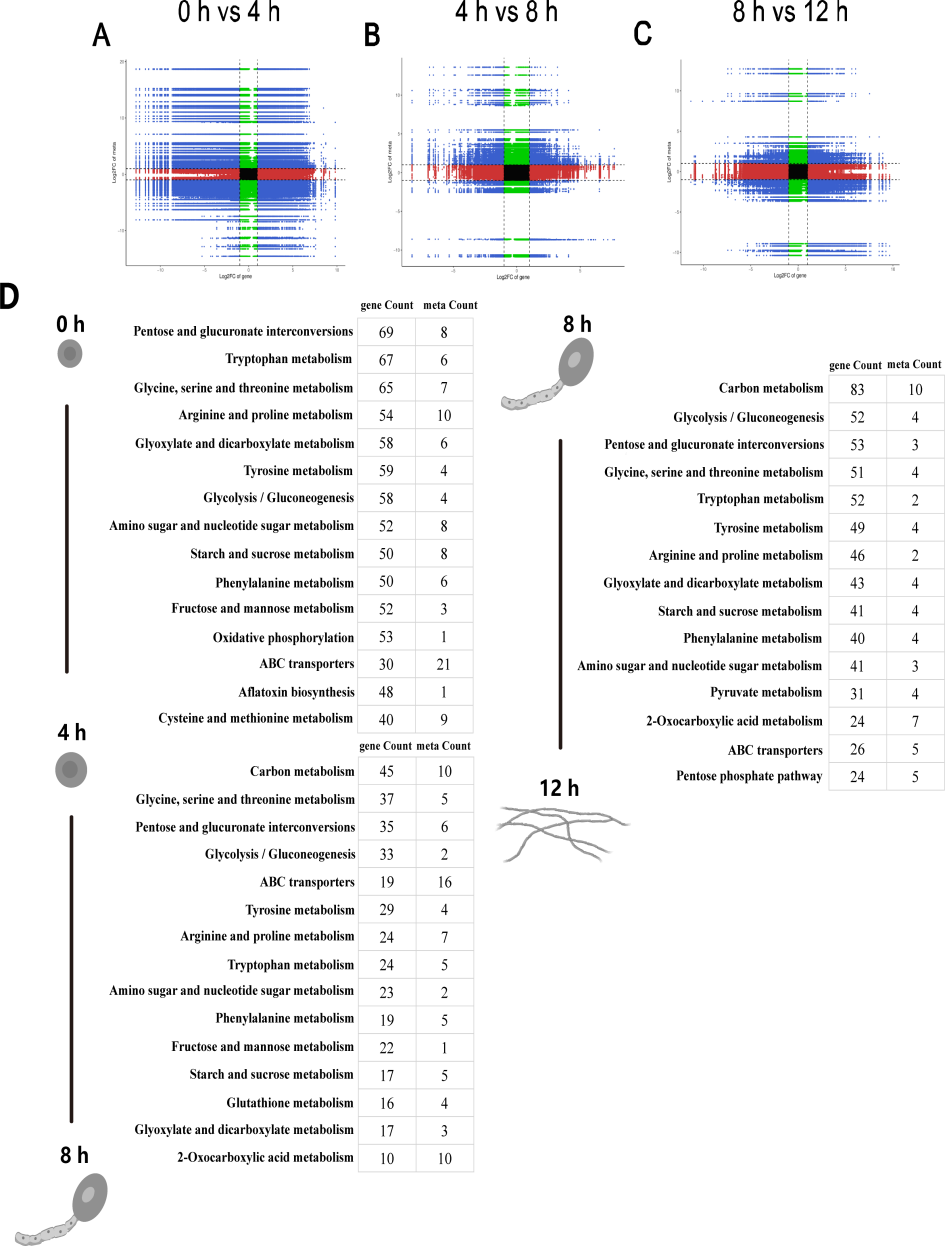
Combined analysis of transcriptomics and metabolomics. The gene expression of two groups was significantly (*P* < 0.05) differentially expressed as fold change >2; The metabolite expression of two groups was significantly (*P* < 0.05) different as VIP >1 and fold change >2. Correlations between different genes and metabolites at 0 h–4 h (A), 4 h–8 h (B), and 8 h–12 h (C) are represented. The x-axis indicates log2FC of genes, and the y-axis indicates log2FC of metabolites. (D) Top 15 KEGG enrichment pathways of 0 h–4 h, 4 h–8 h, and 8 h–12 h are represented.

Correlation analysis was performed on the DEGs and differential metabolites in each group, and Pearson’s correlation coefficients were calculated for correlations between genes and metabolites (Fig. 4A through C). The nine-quadrant diagram shows differences in genes and metabolites with a strong correlation (Pearson coefficient greater than 0.8) for each group being compared. The diagram is divided into nine sections (1–9 from left to right and from top to bottom), with quadrants 3 and 7 representing genes and metabolites that consistently show changes in expression and regulatory patterns. This means that these genes and metabolites are consistently related and may have a positive influence on each other’s activity. The results showed a high consistency between DEGs and differential metabolites in different germination time periods, and the number of DEGs and differential metabolites with consistency across different time periods was slightly lower than that of non-consistent DEGs and differential metabolites. Also, the total number of DEGs and differential metabolites was greater in the early stage of germination than in the later and middle stages. Figure 4D shows the combined KEGG annotation results of DEGs and differential metabolites during different stages of *A. flavus* conidia germination. The results of KEGG annotation mainly focused on secondary metabolite, amino acid metabolism, carbon metabolism, and purine metabolism. Several different pathways included oxidative phosphorylation; cysteine and methionine metabolism; 2-oxocarboxylic acid metabolism; and alanine, aspartate, and glutamate metabolism at the early stage of germination (0 hour vs 4 hours), glutathione metabolism and pyrimidine metabolism at the middle stage of germination (4 hours vs 8 hours), and the pentose phosphate pathway at the late stage of germination (8 hours vs 12 hours).

### Effects of different carbon sources on conidia germination

**Fig 5:**
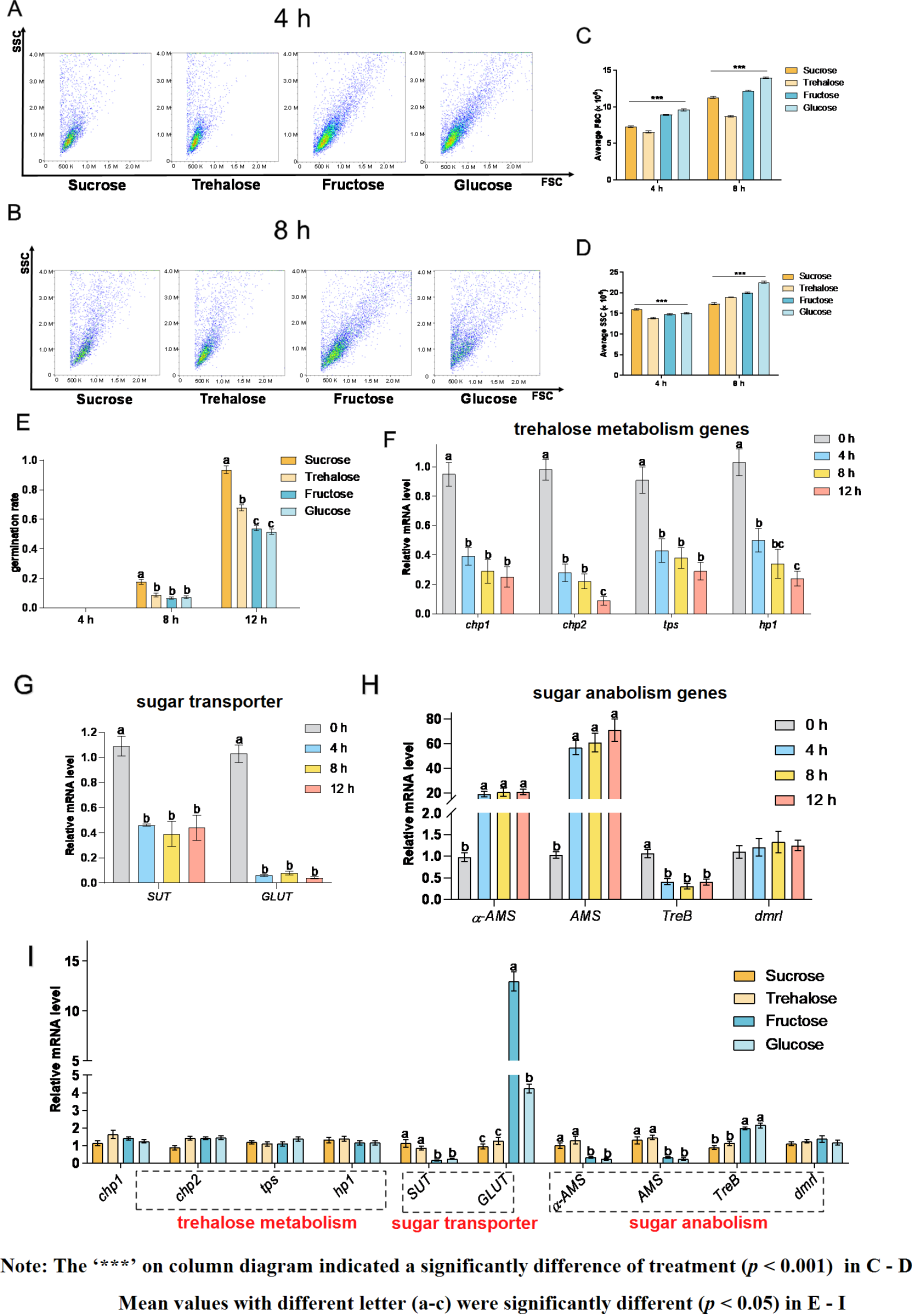
Effects of different carbon sources on *A. flavus* conidia germination and related genes. The size and complexity of conidia were measured by flow cytometry under different carbon sources at 4 hours (A) and 8 hours (B). The x-axis indicates FSC, and the y-axis indicates SSC. Average FSC and SSC parameters of 10,000 conidia were analyzed at 4 hours (C) and 8 hours (D), respectively. The means and standard errors of duplicate samples were plotted (*n* = 3). Mean values with *** were significantly different (*P* < 0.001). (E) The germination rate under different carbon sources and different states. Real-time quantitative PCRs of different stages of conidia with trehalose metabolism genes (F), sugar transporter genes (G), and glucose anabolism genes (H) are shown. (I) Real-time quantitative PCR of 8-h conidia under different carbon sources. The means and standard deviation of duplicate samples were plotted (*n* = 6). Mean values with different letters were significantly different (*P* < 0.05).

Transcriptomic data revealed that genes related to glycolysis / gluconeogenesis and fructose and mannose metabolism changed significantly during conidia germination, with 110 genes significantly changed during 0–4 hours, 55 genes significantly changed during 4–8 hours, and 84 genes significantly changed during 8–12 hours. Therefore, further study is required to evaluate different carbon sources in relation to conidia germination. It could be seen from Fig. 5A through C that in terms of conidia size, the results at 4 hours and 8 hours were consistent. Both fructose and glucose (monosaccharide group) were higher than sucrose and trehalose (disaccharide group). For the SSC of conidia (Fig. 5D), compared with other sugars, sucrose significantly increased the SSC of conidia (conidia in different stages) at 4 hours, and trehalose significantly reduced the SSC of conidia at 4 hours; after culturing for 8 hours, the effect of different sugars on the SSC of conidia was different from that at 4 hours. The complexity of conidia in sucrose was significantly lower than that in trehalose, fructose, and glucose, and the complexity of conidia in glucose was significantly increased over that of other groups. Figure 5E showed that four kinds of sugars had different effects on conidia germination. Compared with other sugars, sucrose significantly improved the germination rate at 8 hours, while trehalose, fructose, and glucose showed no significant differences. Moreover, trehalose and sucrose (as disaccharides) significantly promoted the germination rate compared with fructose and glucose (as monosaccharides) at 12 hours.

Some DEGs in transcriptomic data were verified by qPCR in this study. As shown in Fig. 4F, genes related to trehalose synthesis (*chp1, chp2, tps,* and *hp1*) were gradually downregulated over time. The stable synthesis of trehalose is crucial for maintaining stress resistance, and as conidia germinated, the demand for trehalose gradually decreased. Figure 4G shows that the expressions of sucrose transporter gene (*SUT*) and glucose transporter gene (*GLUT*) were highest at the dormant stage, and the expression of the sugar transporter gene (*SUT*) gradually decreased as the conidia germinated; from Fig. 4H, it was revealed that the expression of genes related to polysaccharide synthesis (*α-AMS* and *AMS*) was lowest at the dormant stage and increased significantly at 4 hours. As germination progressed, the expression of these genes did not change significantly. The qPCR results of 8-h conidia in different carbon sources are shown in Fig. 5I. The expression of polysaccharide synthase genes (*α-AMS* and *AMS*) and the sucrose transporter gene (*SUT*) in sucrose and trehalose was significantly higher than that of fructose and glucose; the expression of glucose transporter (*GLUT*) and trehalose synthesis-related genes (*TreB*) in sucrose and trehalose was significantly lower than that of fructose and glucose. These results revealed that disaccharides may promote the germination of *A. flavus* conidia by increasing the expression of polysaccharide synthase genes.

### Effects of different nitrogen sources on conidia germination

**Fig 6:**
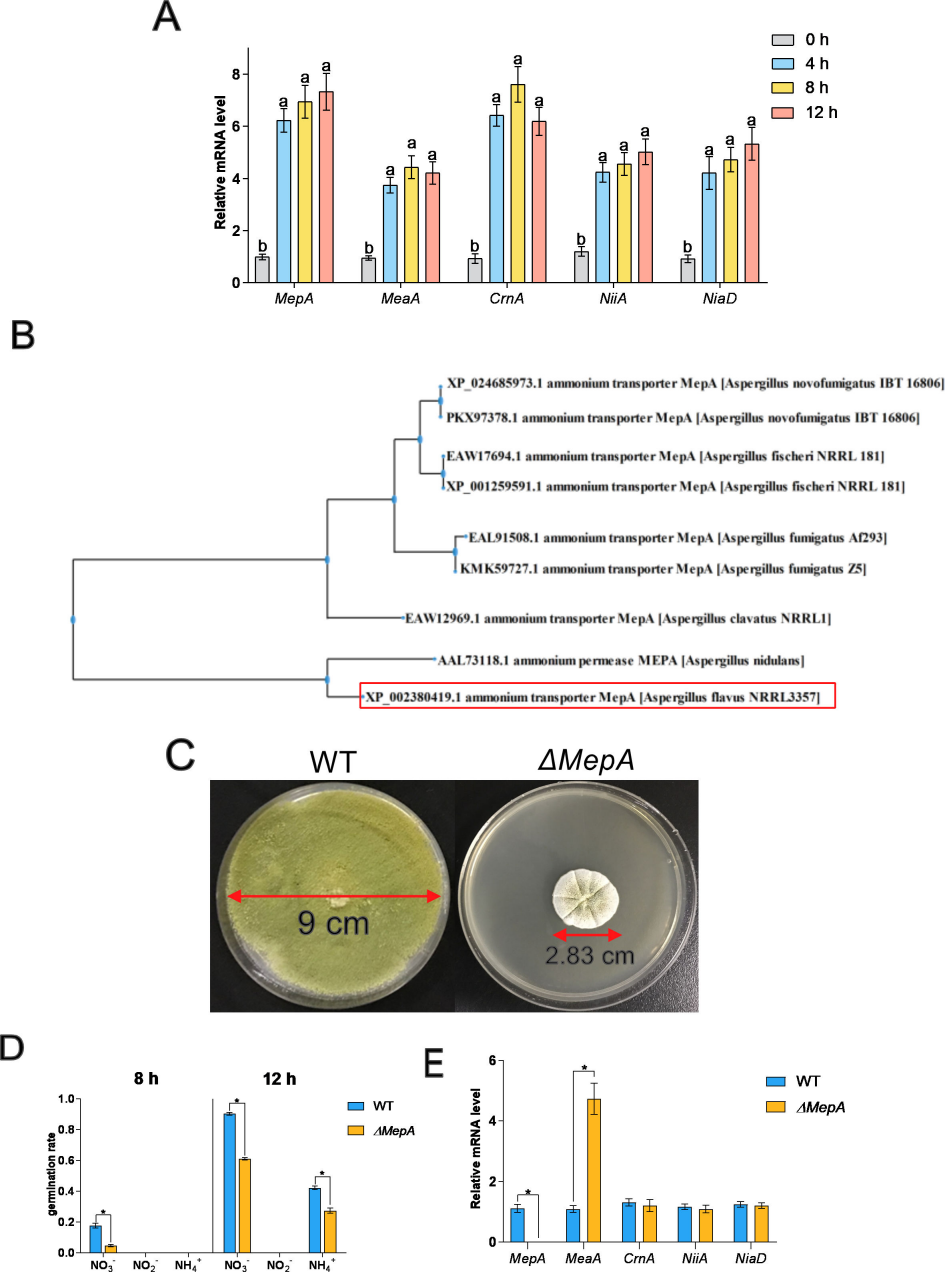
Effects of different nitrogen sources on *A. flavus* conidia germination and related genes. (A) Real-time quantitative PCR of 8-h conidia under different nitrogen sources. The means and standard deviation of duplicate samples were plotted (*n* = 6). Mean values with different letters were significantly different (*P* < 0.05). (B) *MepA* evolutionary tree. (C) Morphological differences between the wild-type and ΔMepA. (D) Germination rate under different nitrogen sources and different states of the wild-type and ΔMepA. (E) Real-time quantitative PCR of 8-h conidia of ΔMepA and the wild-type. The means and standard deviation of duplicate samples were plotted (*n* = 6). Mean values with * were significantly different (*P* < 0.05).

According to our transcriptomic data, the genes related to nitrogen metabolism changed significantly during conidia germination, with 19 genes significantly changed during 0–4 hours, seven genes significantly changed during 4–8 hours, and 12 genes significantly changed during 8–12 hours. Ammonium and methylammonium enter cells through active transport systems, which constitute a transmembrane protein family (ammonium transporters) and have been identified in fungi, bacteria, plants, and animals (36). In order to study the expression of ammonium transporter genes during germination, qPCR was used to detect the expression of related genes (*MepA*, *MeaA*, *CrnA*, *NiiA*, and *NiiD*) (Fig. 6B). During the initial stage (0–4 hours), the expressions of the ammonium transporter genes (*MepA* and *MeaA*), nitrate transporter gene (*CrnA*), nitrate reductase gene (*NiiA*), and nitrite reductase gene (*NiiD*) were significantly upregulated, and thus the expression of these genes was not significantly regulated over time. Compared to dormant conidia, *MepA* was about fivefold upregulated, *MeaA* was about threefold upregulated, *CrnA* was about sevenfold upregulated, and nitrate reductase gene (*NiiA*) and nitrite reductase gene (*NiiD*) were about fourfold upregulated. The results indicated that these genes play a crucial role at the initial stage of germination, but the expression levels did not change over time. Both *MepA* and *MeaA* belong to the ammonium transporter family. Several gene sequences of high homology with the *A. flavus* ammonium transporter were downloaded from the NCBI. Through the evolutionary tree (Fig. 6C), the *MepA* in *A. flavus* was highly homologous to *A. nidulans* and was less homologous to common *Aspergillus* species such as *A. fumigatus*. To study the function of ammonium transporter genes (*MepA* and *MeaA*) in the process of *A. flavus* conidia germination, gene knockout of these two genes was performed. A *ΔMepA* mutant was obtained, but no *ΔMeaA* mutants were viable. A comparison between *ΔMepA* and the wild-type cultured for 7 days is shown in Fig. 6D. The morphology of *ΔMepA* was significantly different from that of the wild-type, with a shorter frontal hyphae and white edges. The colonies showed a wrinkled appearance, and the color of the colonies was slightly brown. The overall colony was significantly smaller (with the diameter of 2.83 cm) than that of the wild-type (with the diameter of 9.00 cm). Since *MepA* is mainly involved in nitrogen metabolism of *A. flavus* conidia, this experiment used equal amounts of nitrate, nitrous acid, and ammonium as the only nitrogen sources to observe the difference in the germination rate between *ΔMepA* and the wild-type. Figure 6E revealed that compared to the wild-type, the germination rate of *ΔMepA* was significantly lower with different nitrogen sources. At 8 hours, the germination rate of wild-type was 17.70%, 0, and 0 in NO_3_^-^, NO_2_^-,^ and NH_4_^+,^ respectively; the germination rate of *ΔMepA* was 4,65%, 0, and 0 in NO_3_^-^, NO_2_^-,^ and NH_4_^+,^ respectively. At 12 hours, the germination rate of wild-type was 90.30%, 0, and 42.27% in NO_3_^-^, NO_2_^-,^ and NH_4_^+,^ respectively; the germination rate of *ΔMepA* was 61.01%, 0, and 27.30% in NO_3_^-^, NO_2_^-,^ and NH_4_^+,^ respectively. According to the transcriptomic data, qPCR was used to detect the relative mRNA level of *MeaA*, *CrnA*, *NiiA,* and *NiaD* in the nitrogen metabolism pathway at 8 hours (Fig. 6F). The expressions of *CrnA*, *NiiA,* and *NiaD* were not significantly different between the two strains, but the expression of *MeaA* was significantly upregulated in the *ΔMepA* strain, which may have been due to the compensatory metabolism of the ammonium transporter; in the absence of *MepA*, the expression of *MeaA* is highly upregulated, to maintain normal germination of *A. flavus* conidia.

## DISCUSSION

The process of conidia germination is generally considered to have three different stages: dormant conidia, isotropic growth, and polarized growth (37). Our study demonstrated that at the transcription level, major changes occurred in the early germination of *A. flavus* conidia (0 hour vs 4 hours), with 5,888 DEGs and 264 differential metabolites. Transcriptional and metabolic processes contained the most differential genes between dormant conidia and 4-h conidia, which may infer that the conidia were transferred from a relatively static, dormant stage to an active state of germination, and during germination, it was necessary to restore and increase metabolic activities, including respiration, DNA synthesis and mitosis, cell wall synthesis, and RNA and protein biosynthesis. A study in *A. niger* (38) using a whole-genome microarray reported that in the process of conidia germination, the main transcriptional changes occurred in the first 2 hours. However, the number of detected genes was only 4,000, far lower than the 11,000 genes in our study. Another study (39) revealed that compared with the subsequent germination stage of *A. fumigatus*, the initial stage of conidia germination had the most significant changes. Our data revealed that in the energy process, the mRNA encoding enzymes of the TCA cycle, glycolysis/gluconeogenesis, and the pentose phosphate pathway were highly expressed in the initial stage of conidia germination, while they were absent in dormant conidia. The gluconeogenesis pathway could be initiated by fatty acids, and fatty acids could enter the gluconeogenesis pathway. The high Rich factor of the degradation and metabolism of fatty acids in the 0-hour vs 4-hour group also demonstrated that gluconeogenesis may play an important role in the survival and germination of conidia through the use of stored lipids. These results were highly consistent with those of another study (40).

Previous studies have shown that dormant conidia are resistant to extreme environments (41). Conidia could survive and germinate in the extreme environments of dehydration, extreme temperature, and osmotic pressure changes due to its three-layer cell wall and special internal structure (10). Most *Aspergillus* dormant conidia start to germinate when they detect the presence of nutrients such as sugar, inorganic salts, and nitrogen sources, and thus the germination period varied between culture conditions and *Aspergillus* strains. Therefore, studying the effects of these nutrients on conidia germination was crucial. Our metabolomics studies found that there were significant differences in the composition of different sugars during different stages of conidia germination, and the fructose content was significantly higher than that of glucose. Also, we found that the expression of genes related to trehalose synthesis was significantly downregulated at the initial stage of germination, while the changes in these genes tend to slow down over time. A recent study (42) demonstrated that the sugar metabolism component of carbon metabolism during the germination process of *Aspergillus* mainly involves the synthesis, metabolism, and transport of polysaccharides. Other studies in *A. nidulans* and *A. fumigatus* (43, 44) reported that trehalose played an important role in the germination of *Aspergillus* conidia. Some proteins related to the trehalose biosynthesis pathway could stabilize the *Aspergillus* cell wall, and trehalose will be rapidly consumed during conidia germination. The results revealed that downregulation of the trehalose synthesis gene may be an important indicator for *A. flavus* to germinate, and the large amount of trehalose in resting conidia was also an important marker for maintaining the stability of dormant conidia. Furthermore, as a disaccharide, trehalose could be decomposed into two glucose molecules at the initial stage of germination, providing energy for the germination of *A. flavus*. Besides, our study also found that during the germination of *A. flavus* conidia, the mRNA levels of sucrose transporter (SUT) and glucose transporter (GLUT) were also significantly downregulated at the initial stage of germination, which indicated that during the dormancy process of conidia, genes related to sugar metabolism are highly expressed to make basic preparations for germination. The downregulation of the expression of sugar transporter-related genes also indicated that the main nutrients in the initial stage of germination were the stored nutrients in dormant conidia. Unlike the sugar transporter genes, polysaccharide synthesis genes were highly expressed in the early stages of conidia germination, with no significant difference as the time increased. The reason may be that the germination process of *A. flavus* conidia was accompanied by the growth of the cell wall, cytoskeleton, and other macromolecular structural materials, which all require the participation of polysaccharides; therefore, expression of polysaccharide synthase genes was significantly upregulated at the initial stage. The germination rate of *A. flavus* conidia differed significantly with the use of different carbon sources. A similar study (45) was performed in other *Aspergillus* species. When xylose was used as the only carbon source, the conidia germination rate of *A. niger* was significantly lower than that of glucose. In another study (46), the conidia germination rate of *A. niger* showed a significant difference when xylose, glucose, cellobiose, glycerol, and sugar-free medium were used. Our study showed that sucrose was more suitable for the germination of *A. flavus* conidia than trehalose, glucose, and fructose. Moreover, the conidia germination rates of trehalose and sucrose (as disaccharides) were significantly higher than those of glucose and fructose (as monosaccharides) at the later stage of germination. Interestingly, the results of flow cytometry showed that, in contrast to the trend in the conidia germination rate, the cell volume of the glucose group and fructose group as monosaccharides was significantly larger than that of the trehalose group and sucrose group during the early stage of *A. flavus* conidia germination, which may be explained by the difference in the osmotic pressure in the culture medium caused by the composition of different sugars. The results also showed that the water absorption of conidia was not positively correlated with the conidia germination rate in *A. flavus*.

Our study used Czapek–Dox medium, in which sodium nitrate was the only nitrogen source. When germination started, several genes involved in nitrogen metabolism were regulated. For example, *NR*, *rt*, and *nit6* were used to increase the transcriptional levels of nitrate to ammonia, and through a series of biological processes, amino acids were synthesized, which were then assembled into various active proteins. In this study, it was shown that when *A. flavus* conidia use nitrate as the only nitrogen source, the related pathways of nitrogen metabolism and the expression of nitrogen metabolism-related genes were significantly different. The result of qPCR was consistent with the trend in the transcriptome analysis; at the initial stage of conidia germination, several nitrogen metabolism-related genes were significantly upregulated, including ammonium transporter, nitrate reductase, and nitrous acid reductase, while these genes were not significantly regulated at the later stage of germination. Studies have shown that the response mechanisms of filamentous fungi such as *A. nidulans* and *Neurospora crassa* (47) to different types of nitrogen sources and different concentrations of nitrogen sources in culture media are regulated at multiple levels, with the most important being transcriptional level regulation. Ammonium salts are considered a high-quality nitrogen source, while nitrate is considered a stimulating nitrogen source for filamentous fungi. Our study demonstrated that when different inorganic nitrogen sources were used as the only nitrogen source, the conidia germination rate in nitrate was significantly higher than that in nitrite and ammonium. The results of another study in *Penicillium oxalicum* (48) were consistent with that of ours. Ammonium and similarly methyl ammonium enter cells through active transport systems, which have been identified as the transmembrane protein family in fungi, bacteria, and plants (49). These systems are named ammonium transport carriers. Ammonium transporter genes are relatively conserved and mainly include three categories: amtB, Mep, and RH proteins. Among them, Mep is mainly present in fungi such as *yeast*, while amtB is mainly distributed in bacteria and archaea. In our study, *ΔMeaA* of *A. flavus* could not grow normally, while *ΔMepA* of *A. flavus* could grow in a normal medium, with the germination rate significantly lower than that of the wild-type, and the results for the germination rate were consistent in the culture medium with ammonium salt, nitrate, and nitrite as the only nitrogen sources. Furthermore, the gene expression level of *ΔMepA* and wild-type conidia after culturing for 8 hours was significantly different only in *MeaA*, and the other nitrate reductase and nitrous acid reductase-related genes showed no significant difference, which indicated that *MepA* could partially replace the function of *MepA*, but *MepA* was indispensable in *A. flavus*. When *MepA* was missing in the strain, the mRNA expression level of *MeaA* showed compensatory upregulation. Previous research (50) demonstrated that *MepA* and *MeaA* in *A. niger* have different affinities for different ammonium sources. Deletion of *MeaA* and *MepA* made the strain unable to grow at ammonium concentrations below 10 mM. At the same concentration, the growth of the *ΔMeaA* decreased, while *ΔMepA* exhibited normal growth.

### Conclusion

**Fig 7:**
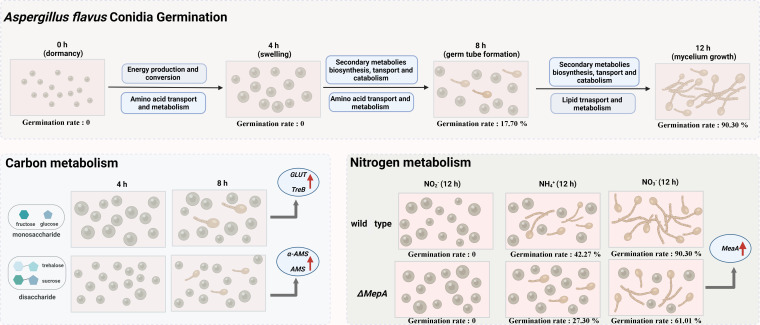
Summary of *A. flavus* conidia germination.

The main results of this study are shown in Fig. 7. Briefly, the *A. flavus* conidia completed germination in 12 hours. Both transcriptomic and metabolomic data showed the greatest changes from 0 hour to 4 hours. Conidia germination in different carbon sources demonstrated that compared with polysaccharides, monosaccharides significantly increased the size of conidia and significantly decreased the germination rate of conidia. Conidia germination in different nitrogen sources demonstrated that nitrate was more suitable for *A. flavus* conidia germination than ammonium salt and nitrite. Besides, *MeaA* and *MepA* played a vital role in ammonia transport and metabolism during *A. flavus* conidia germination.

The results of this study provide a theoretical basis for inhibition of *A. flavus* conidia germination. Also, exploring the effects of different carbon and nitrogen sources on the germination of *A. flavus* conidia could improve our understanding of why different food and feed crops are polluted by aflatoxins to a different extent. However, the actual composition of food and feed crops is far more complex, and further studies should be performed to investigate the germination of *A. flavus* conidia in practical situations.

## Data Availability

The sequencing data generated in this study have been deposited in NCBI’s Sequence Read Archive database (SRA, http://www.ncbi.nlm.nih.gov/Traces/sra_sub/sub.cgi) and are accessible through SRA series accession number PRJNA978970.

## AFTER EPUB

*[This article was published on 23 July 2024 with errors in the Acknowledgments. The funding information in the Acknowledgments was corrected in the current version, posted on 31 July 2024.]*

## SUPPLEMENTAL MATERIAL

Supplemental material spectrum.00108-24-s0001.docx Tables S1 to S3.

ASM does not own the copyrights to Supplemental Material that may be linked to, or accessed through, an article. The authors have granted ASM a non-exclusive, world-wide license to publish the Supplemental Material files. Please contact the corresponding author directly for reuse.
