# Supplementary material for: Study on the metabolic changes and regulatory mechanism of Aspergillus flavus conidia germination
Source: Microbiol Spectr. 2024 Jul 23;12(9):e00108-24. doi: 10.1128/spectrum.00108-24 (PMC11370259; doi:10.1128/spectrum.00108-24)
Supplement: Supplemental material — Tables S1 to S3. [file spectrum.00108-24-s0001.docx]

**Table S1.** Primers used in constructing *ΔMepA* strain

| Primers (for construct *ΔMepA*) | Sequence (5′-3′) |
| --- | --- |
| Above F | GAGACTGTCAGCAAACCCTA |
| Above R | GAAGTAGCCGAGCAATGAGCCGTCGAAACAATAACCC |
| Middle F | GGGTTATTGTTTCGACGGCTCATTGCTCGGCTACTTC |
| Middle R | AGCTACCATGCGACCTTCGCCTTCCTCCACAACACTC |
| Below F | GAGTGTTGTGGAGGAAGGC CCGATTACTTGTCTATGAT |
| Below R | GGATTTTCTAAGTCTTTCG |
| Primers (for validate *ΔMepA*) | Sequence (5′-3′) |
| *ΔMepA* Above F | CCTCCAACAGAGTTTCCA |
| *ΔMepA* Above R | GGTCGTATCAAGGTCGTG |
| *ΔMepA* Above-Middle F | TTGGGCATAACTAATAAAGG |
| *ΔMepA* Above-Middle R | CGATGTAGGGACCGAGAC |
| *ΔMepA* Below-Middle F | GGTCTCGGTCCCTACATC |
| *ΔMepA* Below-Middle R | AATCCTAAACGCCAGTTC |
| *ΔMepA* Below F | GGTCTCGGTTTCCTTTATT |
| *ΔMepA* Below R | CCGTCAAGCATCATTTCG |

| Primers | Sequence (5′-3′) | Primers | Sequence (5′-3′) |
| --- | --- | --- | --- |
| *GAPDH* F | TCTTCTGGGTAGCAGTGTAGGAG | *DMRL* F | CGTGCCTGTCATTTTTGGGG |
| *GAPDH* R | ACAAGGAATACAAGACCGACATC | *DMRL* R | CCATGAGGGCCTGTTACTGA |
| *tp1* F | ATTGAGCTTTGTACGGGCGA | *MepA* F | TGTTTGCGTGCATTACCGTG |
| *tp1* R | TCGCGCACCTAATTTGACCT | *MepA* R | CGTTAAAGCCAAACCAGCCC |
| *tp 2* F | GGATCGTTACATGCGCCAAC | *MeaA* F | ATATCCGGCCTTTCTCGTGT |
| *tp2* R | GATGTTAGAGCCATCGCGGA | *MeaA* R | CAGGCCGAAGTTACGAAGGT |
| *hp1* F | TTCCGGATACCACCCAGGAT | *CrnA F* | CCTCCCCACCTGAGTCCATA |
| *hp1* R | AAATGCTATCGCCGCGTTTC | *CrnA* R | GTAGAAGTCGCCGAGGATGG |
| *tps* F | GTTTGGAGGTGTCCGTCGAT | *NiiA* F | CCAAACTGAAACTGCTGGGC |
| *tps* R | AGACTTGCGCCTCCAATTCA | *Niia* R | GCCAGATCATCGCCGTTTTC |
| *SUT* F | CGCTTTCGTCTGGATTGCTG | *NiaD* F | CGCCGTTATTTGGTCGTACT |
| *SUT* R | CTGATTCCTGTTGGTGGGCT | *NiaD* R | TGTTGAGCCACCTCTCACAG |
| *GLUT* F | AAAATGGGCGATCACGGCTA | *SS-CAT* F | ACGCTTGTAGTTCCGATGCT |
| *GLUT* R | GAGACCATTTGGAGGCCGAA | *SS-CAT* R | TGTGAAGGTCGCTACGTCTG |
| *TreB* F | CAAGGACTGGACAGACGACC | *M-CAT* F | GATCGAAGCCAAACTTCAGC |
| *TreB* R | TCCTCCGGGAACCACAAATG | *M-CAT* R | CTCCAAGCTCGTCAAGTTCC |
| *α-AMS* F | ATAAGGGTGCCGACGGTTTT | *CAT* F | ACCGGTGGTACTGATTCTGC |
| *α-AMS* R | TCTGGCATCTCTCCGACTGA | *CAT* R | CGAGACACTGGCTCATTTCA |
| *AMS* F | AGTACTGGCTTGACATGGGC | *CAT2* F | TCAATCAGATGGAGCCTGTG |
| *AMS* R | CGTAGTCCTGGACGATGCTC | *CAT2* R | GCCGGGTAGTAAACACTCCA |

**Table S2.** Primers used in qPCR

**Table S3.** The KEGG pathways and fold change of interested DEGs

| **Gene ID** | | | **Description** | **Log_2_FC (0 - 4 h)** | | **Log_2_FC**  **(4 - 8 h)** | **Log_2_FC**  **(8 – 12h)** |
| --- | --- | --- | --- | --- | --- | --- | --- |
| **Pentose phosphate pathway** | | | | |  |  |  |
| G4B84_006344 | | transketolase TktA | | | 1.391 | -1.275 | 1.533 |
| G4B84_004006 | 6-phosphofructokinase alpha subunit | | | | 0.494 | -1.206 | 1.198 |
| G4B84_002360 | ribokinase | | | | 3.168 | -3.534 | 2.477 |
| G4B84_006151 | ribulose-phosphate 3-epimerase | | | | 0.119 | -1.186 | 0.872 |
| **Citrate cycle (TCA cycle)** | | | | |  |  |  |
| G4B84_001674 | | | pyruvate dehydrogenase complex, dihydrolipoamide acetyltransferase | 2.745 | | -1.046 | 1.289 |
| **Glycolysis / Gluconeogenesis** | | | |  |  |  |  |
| G4B84_000363 | | | betaine-aldehyde dehydrogenase, putative | | 3.481 | -2.643 | 6.964 |
| G4B84_009346 | | | glyceraldehyde-3-phosphate dehydrogenase, putative | | 2.852 | 6.208 | -6.094 |
| G4B84_001674 | | | pyruvate dehydrogenase complex, dihydrolipoamide acetyltransferase | | 2.745 | -1.046 | 1.289 |
| G4B84_010518 | | | pyruvate decarboxylase, putative | | 4.402 | -2.075 | 4.146 |
| G4B84_007664 | | | alcohol dehydrogenase, putative | | -3.494 | -2.112 | 1.735 |
| G4B84_009788 | | | phosphoglycerate kinase PgkA, putative | | 1.893 | -1.107 | 2.327 |
| **Starch and sucrose metabolism** | | | | |  |  |  |
| G4B84_006407 | | | glycogen phosphorylase GlpV/Gph1, putative | | 1.386 | -2.277 | 2.053 |
| G4B84_003073 | | | glucoamylase precursor, putative | | -3.39 | -1.044 | -1.211 |
| G4B84_006002 | | | beta-glucosidase, putative | | -1.76 | -4.118 | 4.026 |
| G4B84_009541 | | | alpha,alpha-trehalose-phosphate synthase subunit Tps2, putative | | 0.248 | -1.187 | 1.566 |
| **Fructose and mannose metabolism** | | | | |  |  |  |
| G4B84_009024 | | | sorbitol/xylitol dehydrogenase, putative | | 1.827 | -1.034 | -0.34 |
| G4B84_000483 | | | L-arabinitol 4-dehydrogenase | | -0.343 | 1.365 | -6.912 |
| G4B84_004006 | | | 6-phosphofructokinase alpha subunit | | 0.494 | -1.206 | 1.198 |
| G4B84_003734 | | | mannose-1-phosphate guanylyltransferase | | 3.456 | -1.031 | 2.295 |
| G4B84_006105 | | | short chain dehydrogenase/oxidoreductase, putative | | -4.653 | -1.321 | -2.328 |
| G4B84_000015 | | | short-chain dehydrogenase, putative | | -4.134 | -1.145 | -0.052 |
| G4B84_000079 | | | short-chain dehydrogenase, putative | | 3.065 | -1.642 | -0.81 |
| G4B84_007868 | | | short-chain dehydrogenase, putative | | -3.463 | 2.116 | -1.049 |
| G4B84_003431 | | | oxidoreductase, short chain dehydrogenase/reductase family | | 1.739 | 1.061 | -0.623 |
| **Propanoate metabolism** | | | | |  |  |  |
| G4B84_000272 | | | methylmalonate-semialdehyde dehydrogenase, putative | | -0.762 | 1.08 | -2.931 |
| G4B84_002631 | | | 2-oxoisovalerate dehydrogenase complex alpha subunit, putative | | -1.076 | 2.171 | -1.172 |
| **Arginine and proline metabolism** | | | | |  |  |  |
| G4B84_000363 | | | betaine-aldehyde dehydrogenase, putative | | 3.481 | -2.643 | 6.964 |
| G4B84_004218 | | | amine oxidase, putative | | 1.496 | -3.044 | 2.111 |
| G4B84_007377 | | | putative polyamine oxidase | | -3.322 | 3.063 | -4.519 |
| G4B84_011570 | | | flavin-containing amine oxidase, putative | | 1.908 | -1.607 | -0.558 |
| G4B84_004019 | | | amidase, putative | | 2.937 | 1.668 | 0.013 |
| G4B84_007298 | | | glutamyl-tRNA(gln) amidotransferase subunit A, putative | | 2.071 | -1.664 | 4.692 |
| G4B84_003617 | | | aromatic-amino-acid aminotransferase, putative | | -1.829 | 2.021 | -1.202 |
| **ABC transporters** | | | | |  |  |  |
| G4B84_006423 | | | ABC multidrug transporter, putative | | 4.997 | -3.095 | 0.985 |
| G4B84_008991 | | | ABC multidrug transporter, putative | | -2.228 | 2.237 | -2.452 |
| G4B84_009858 | | | hypothetical protein AFLA_068510 | | 1.437 | -2.303 | 1.203 |
| G4B84_007753 | | | ABC transporter, putative | | 5.438 | -1.055 | 2.192 |
| G4B84_011881 | | | ATP-binding cassette transporter, putative | | 1.206 | -3.216 | 3.89 |
| **Oxidative phosphorylation** | | | | |  |  |  |
| G4B84_002955 | | | cytochrome c oxidase assembly protein cox15 | | 1.394 | 1.395 | 0.442 |
| G4B84_002721 | | | NADH-ubiquinone oxidoreductase 64 kDa subunit, putative | | 1.098 | -1.648 | 2.747 |
| **Aflatoxin biosynthesis** | | | | |  |  |  |
| G4B84_007652 | | | methylmalonyl-CoA decarboxylase, alpha subunit, putative | | -2.609 | 0.289 | -0.976 |
| G4B84_005809 | | | aflC / pksA / pksL1 / polyketide synthase | | 1.946 | -0.574 | 0.072 |
| G4B84_003994 | | | short chain oxidoreductase (CsgA), putative | | -1.399 | -0.137 | -0.55 |
| G4B84_004954 | | | toxin biosynthesis ketoreductase, putative | | 5.021 | -0.61 | 2.009 |
| G4B84_005807 | | | Nor-1 | | 1.082 | -0.243 | 1.082 |
| G4B84_010450 | | | hydroxyacyl dehydrogenase, putative | | 4.16 | -1.244 | 2.602 |
| G4B84_003628 | | | short chain type dehydrogenase, putative | | 2.016 | -0.724 | 3.414 |
| G4B84_007041 | | | cytochrome P450 oxidoreductase, putative | | 2.278 | 0.071 | -0.337 |
| G4B84_002451 | | | cytochrome P450, putative | | 6.658 | 0.267 | -0.677 |
| G4B84_011560 | | | glucose-methanol-choline (gmc) oxidoreductase, putative | | 6.866 | -1.307 | 4.781 |
| G4B84_008904 | | | aryl-alcohol dehydrogenase, putative | | -6.586 | 0.973 | -1.631 |
| G4B84_002264 | | | aldo/keto reductase, putative | | -2.127 | 1.198 | -0.339 |
| G4B84_003736 | | | lipase/esterase, putative | | 2.503 | -0.066 | 0.059 |
| G4B84_009404 | | | hypothetical protein AFLA_102120 | | 4.038 | -0.38 | 0.505 |
| G4B84_002149 | | | carboxylesterase, putative | | -6.68 | 1.201 | -0.075 |
| G4B84_004287 | | | methyltransferase, putative | | -4.352 | -0.842 | 0.206 |
| G4B84_000472 | | | O-methyltransferase, putative | | -1.162 | -1.118 | 3.103 |
| G4B84_000091 | | | O-methyltransferase, putative | | 4.867 | -0.682 | 0.306 |
| G4B84_005687 | | | flavonoid 3-hydroxylase, putative | | -3.614 | -0.153 | 0.19 |
| G4B84_006292 | | | cytochrome P450 oxidoreductase OrdA-like, putative | | -7.468 | 3.65 | 0.38 |
| G4B84_007863 | | | cytochrome P450, putative | | 2.612 | -0.362 | 1.012 |
| G4B84_011302 | | | 3-hydroxyacyl-CoA dehydrogenase, putative | | 5.357 | -0.807 | 3.208 |
